# Super-resolution ultrasound quantifying microvascular alterations for early detection of metastatic cervical lymph nodes: a prospective diagnostic study

**DOI:** 10.1038/s41598-025-31523-y

**Published:** 2025-12-06

**Authors:** Jun Zhang, Zhiyu Zhao, Xin Huang, Xingyue Huang, Yao Zhang, Yugang Hu, Qing Deng, Qing Zhou, Qing Zhou

**Affiliations:** Department of Ultrasound Imaging, Renmin Hospital of Wuhan University, No. 99 Zhang Zhidong Road, Wuchang District, Wuhan, 430061 China

**Keywords:** Super resolution ultrasound, Lymph node metastasis, Microvascular imaging, Hemodynamics, Diagnostic performance, Cancer, Diseases, Medical research, Oncology

## Abstract

To evaluate super-resolution ultrasound (SRUS) for characterizing microvascular morphology and hemodynamics in metastatic versus reactive cervical lymph nodes (LNs), with the aim of improving metastatic detection and reducing unnecessary biopsies. In this prospective study, 166 patients with histopathologically confirmed cervical LNs (77 metastatic, 89 reactive) underwent conventional ultrasound and contrast-enhanced SRUS (CE-SRUS) using a commercial US system and SonoVue^®^ microbubbles. Quantitative SRUS parameters vascular density (VD), fractal dimension (FD), flow-weighted vascular density (FWVD), perfusion index (PI), velocity entropy (Vel Entropy), minimum velocity (Vmin) were extracted from whole-LN ROIs. Diagnostic performance was assessed via receiver operating characteristic (ROC) analysis and multivariate logistic regression. Metastatic LNs showed significantly higher VD (0.482 ± 0.073 vs. 0.405 ± 0.168, *p* < 0.001), FD (1.678 ± 0.070 vs. 1.626 ± 0.098, *p* < 0.001), FWVD (1.784 ± 0.592 vs. 1.495 ± 0.813, *p* = 0.013), PI (12.617 ± 2.563 vs. 10.369 ± 5.006, *p* < 0.001), and Vel Entropy (0.922 ± 0.092 vs. 0.796 ± 0.199, *p* < 0.001), but lower Vmin (2.572 ± 2.200 mm/s vs. 2.645 ± 2.800 mm/s, *p* = 0.017) compared to reactive LNs. Univariate ROC top performers included Dir Entropy (AUC = 0.723) and VD (AUC = 0.689). Multivariate analysis identified VD (OR = 1.046, *p* = 0.001), Vmin (OR = 0.525, *p* = 0.003), Velocity Variance (Vel Var) (OR = 1.973, *p* = 0.016), Vel Entropy (OR = 4.674, *p* = 0.042), and PI (OR = 2.481, *p* = 0.018) as independent predictors. The combined model achieved superior diagnostic performance (AUC = 0.813, 95% CI: 0.748–0.879; sensitivity = 76.6%, specificity = 79.8%; *p* < 0.001). SRUS enables non-invasive, high-resolution quantification of microvascular alterations in metastatic LNs. A multivariate model demonstrates excellent discriminative power, demonstrating significant potential to improve preoperative assessment and biopsy guidance in head and neck cancer.

## Introduction

Accurate assessment of cervical lymph node (LN) metastasis is critical for cancer diagnosis, staging, prognosis, and treatment planning, as metastatic status profoundly influences patient survival and management strategies^1,2^. However, the detection of small metastatic foci, particularly micrometastases (< 2 mm), and the differentiation between metastatic and benign reactive lymphadenopathy remain significant clinical challenges^3,4^. While widely available, conventional ultrasound (US) is fundamentally limited by the diffraction barrier, resulting in a spatial resolution of approximately 200 μm. This resolution constraint impedes the visualization of microvascular architecture and hemodynamic alterations indicative of metastasis^5^. Traditional Doppler techniques, such as power Doppler, provide information on larger vessels but fail to overcome this resolution limitation, leading to insufficient depiction of the microvascular network^6,7^. Although CEUS improves the characterization of blood flow features, its resolution remains inadequate for the precise detection of micron-scale microvascular abnormalities associated with metastasis, particularly within LNs^8^.

SRUS represents a revolutionary breakthrough by overcoming the diffraction limit of conventional US. Utilizing microbubbles as point targets for localization and tracking, SRUS achieves spatial resolutions approaching 10 μm^9,10^. This technology enables high-precision mapping of microvascular morphological features (e.g., branching patterns, density) and quantitative assessment of hemodynamic parameters (e.g., flow velocity, direction), providing a comprehensive structural and functional evaluation of tissue microvasculature^11,12^. Emerging evidence supports the feasibility of SRUS for LN assessment. Preliminary studies indicate significant differences (> 60%) in local flow direction irregularities between metastatic and benign LNs, suggesting this as a promising quantitative biomarker for early metastasis detection^13^.

Building on these advances, this study aimed to systematically evaluate the efficacy of SRUS in characterizing microvascular morphology and hemodynamics within cervical LNs to predict the presence of micrometastasis. Specifically, we investigate the diagnostic value of quantitative SRUS-derived parameters, such as vascular irregularity and localized hemodynamic abnormalities, in differentiating malignant from benign lymphadenopathy. This prospective study assesses the potential of SRUS as a non-invasive, high-resolution diagnostic tool for clinical practice, ultimately aiming to enhance precision in the management of patients with head and neck cancer.

## Materials and methods

### Ethical approval

This study was conducted in accordance with the Declaration of Helsinki and received approval from the Ethics Committee of Renmin Hospital of Wuhan University (Approval No.: WDRY2024-K109; Approval Date: June 6, 2024). Written informed consent was obtained from all participants, covering the use of contrast agents and authorization for secondary data usage.

### Study population

**Fig. 1:**
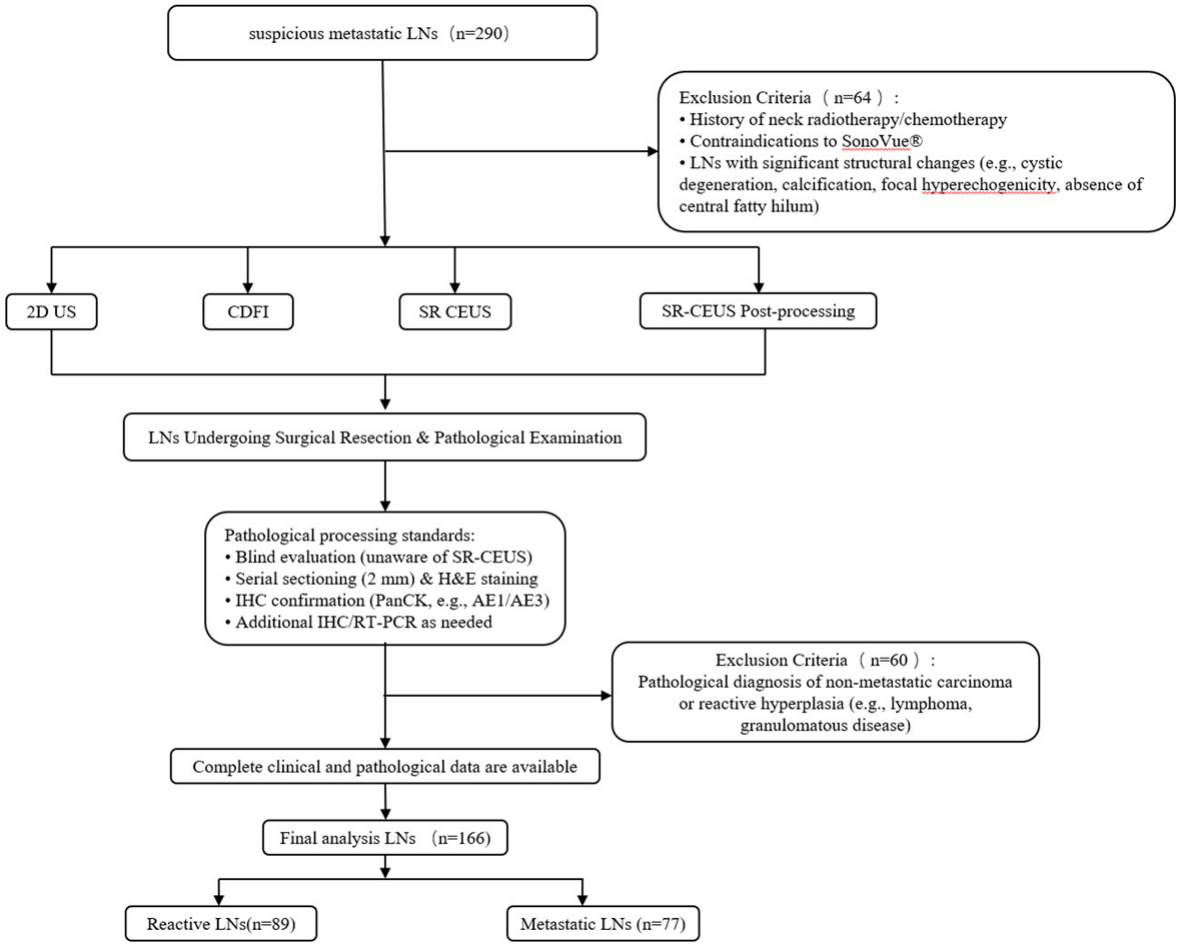
Patient Enrollment and Lymph Nodes (LNs) Selection Flowchart. The diagram outlines the process of screening, enrolling, and excluding patients with suspected cervical LNs metastasis, culminating in the final cohort of 166 histopathologically confirmed LNs included in the analysis.

A total of 290 patients with suspected metastatic cervical LNs were prospectively enrolled between December 2023 and June 2024. All patients underwent surgical LNs resection for histopathological confirmation of nodal status. Pathologists, blinded to SRUS results, processed the LNs, performing serial sectioning at 2-mm intervals followed by Hematoxylin and Eosin (H&E) staining. Suspicious areas were validated using immunohistochemistry (IHC) with AE1/AE3/PanCK antibodies. Additionally, samples underwent IHC or molecular testing (e.g., reverse transcription polymerase chain reaction, RT-PCR) to enhance detection of micrometastases. Inclusion Criteria: LNs meeting the following criteria were included: Availability of complete clinical and pathological data. Absence of contraindications to ultrasound contrast agents. Exclusion Criteria: LNs meeting the following criteria were excluded: Pathological diagnosis other than metastatic carcinoma or reactive hyperplasia (e.g., lymphoma, granulomatous disease). History of prior neck radiotherapy or chemotherapy. Contraindication to the ultrasound contrast agent SonoVue^®^ (sulfur hexafluoride microbubbles; Bracco Imaging S.p.A., Milan, Italy). Presence of significant structural alterations (e.g., cystic degeneration, calcification, focal hyperechogenicity, absent fatty hilum). Ultimately, 166 LNs from these patients met the study criteria and were included in the final analysis. (Fig. 1)

### Imaging protocol

#### Conventional ultrasound

Performed using a Resona A20 Pro ultrasound system (Mindray, Shenzhen, China) equipped with an LM18-5WU linear array transducer (frequency range: 5–18 MHz). Scanning parameters (dynamic range, gain, depth) were optimized according to the manufacturer’s specifications.

##### 2D ultrasound

Assessed lymph node size and morphology. The long-axis view (along the greatest dimension of the LN) was selected to minimize arterial pulsation artifacts. This orientation typically positions the major axis of the node parallel to the skin surface and, often, to the direction of nearby pulsating carotid or vertebral arteries. By aligning the imaging plane this way, the transmitted pulsatile motion from these adjacent major arteries is more likely to cause in-plane, synchronous displacement of the entire node, rather than creating complex, out-of-plane shearing or irregular movements that generate severe artifacts. This facilitates more stable imaging and subsequent accurate microvascular tracking during SRUS acquisition.

##### Color doppler flow imaging (CDFI)

Gain was adjusted to clearly delineate small vessels while avoiding noise artifacts. Probe pressure was minimized. CDFI was used for initial assessment of larger intra-nodal vessels; however, its quantitative data were not included in the subsequent SRUS analysis due to its inferior resolution for microvasculature.

*Super-resolution Contrast Enhanced Ultrasound (SR CEUS)*: Conducted using an SL10-3U transducer (Resona A20 Pro; frequency range: 3–10 MHz, center frequency: 6 MHz). Contrast Agent Administration: SonoVue^®^ (Bracco, Milan, Italy) was administered as a 2.0 mL bolus injection via the antecubital vein, followed by a 5 mL saline flush. Acquisition Parameters: Microvascular imaging mode was employed (mechanical index, MI = 0.116). Depth was standardized to 3 cm. Image acquisition was triggered at peak LNs enhancement. Image Acquisition: Patients were instructed to hold their breath during a 6-second high-frame-rate acquisition (500 frames per second, total 3000 frames). A built-in real-time motion correction algorithm compensated for tissue displacement. A single SRUS microvasculature map was generated after processing the 3000 frames acquired during the 6-second breath-hold. This composite image was saved for offline quantitative analysis. Quality Control: The built-in motion correction algorithm tracked LNs displacement. If correction failed (inter-frame displacement > 0.5 mm), the scan was repeated after a 20-minute interval.

### Quantitative image analysis

Offline quantitative analysis was performed using dedicated SR CEUS processing software (SR CEUS Platform, Mindray Biomedical Electronics Co., Ltd., Shenzhen, China). Real-time visualization provided qualitative flow information, while all quantitative parameters were extracted offline. Region of Interest (ROI) Definition: A fixed ROI encompassing the entire LNs parenchyma was manually delineated. ROIs were defined independently by two radiologists, each with over ten years of experience. Inter-observer agreement was excellent (intraclass correlation coefficient, ICC > 0.85). Parameter Calculation: Based on the enhancement intensity maps and flow velocity maps obtained within the ROI, the software automatically calculated the following hemodynamic parameters:

#### Vascular density (VD)

Percentage of vascular pixels within the ROI, reflecting microvascular richness.

#### Maximum vessel diameter (Dmax)

Estimated maximum vessel diameter based on density distribution.

#### Mean vessel diameter (Dmean)

Estimated mean vessel diameter based on density distribution.

#### Fractal dimension (FD)

Quantifies the complexity and branching pattern of the vascular network; higher values indicate greater complexity.

#### Flow weighted density of the vessel (FWVD)

Sum of signal intensities from vascular pixels within the ROI divided by the total number of pixels within the ROI, reflecting total blood volume.

#### Mean flow velocity (Vmean)

Average blood flow velocity (mm/s) within the ROI.

#### Maximum flow velocity (Vmax)

Maximum blood flow velocity (mm/s) within the ROI.

#### Minimum flow velocity (Vmin)

Minimum blood flow velocity (mm/s) within the ROI.

#### Velocity variance (Vel Var)

Variance of blood flow velocity values within the ROI, reflecting spatial heterogeneity of velocity.

#### Velocity entropy (Vel Entropy)

Entropy of the blood flow velocity distribution; higher values indicate more chaotic/disordered flow.

#### Direction variance (Dir Var)

Variance of blood flow direction vectors within the ROI, reflecting spatial heterogeneity of direction.

#### Direction entropy (Dir entropy)

Entropy of the blood flow direction distribution; higher values indicate more chaotic/disordered direction.

#### Perfusion index (PI)

Product of Vmean and VD, reflecting tissue perfusion level.

### Time-resolved parameter extraction

**Fig. 2:**
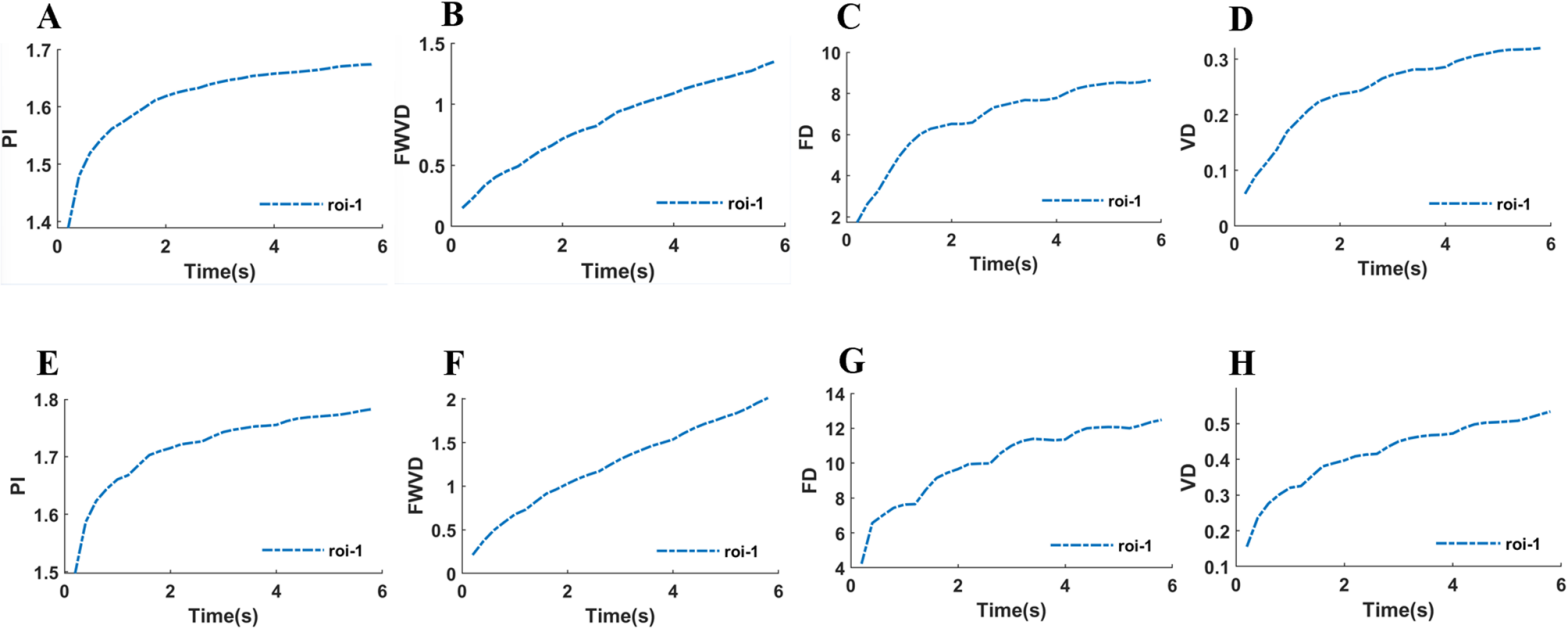
Comparative Super-Resolution Ultrasound (SRUS) Parameter Maps of a Reactive and a Metastatic Cervical Lymph Node. The parameter maps are derived from consecutive 1-second segments of the 6-second acquisition, illustrating temporal variations in Perfusion Index (PI), Flow-Weighted Vascular Density (FWVD), Fractal Dimension (FD), and Vascular Density (VD). The upper row depicts a reactive LN, showing relatively stable and organized values across time segments. The lower row depicts a metastatic LN, demonstrating elevated values and greater spatiotemporal heterogeneity.

In addition to the composite SRUS map generated from the entire 6-second acquisition, the SR CEUS software also computed partial parameter maps by processing consecutive 1-second segments (500 frames per segment). These time-resolved maps were used to visualize the temporal evolution of parameters such as PI, FWVD, FD, and VD, as shown in Fig. 2.

### Statistical analysis

Statistical analyses were performed using IBM SPSS Statistics (Version 26.0; IBM Corp., Armonk, NY, USA) and MedCalc Statistical Software (Version 22.0.1; MedCalc Software Ltd, Ostend, Belgium). Continuous variables are presented as mean ± standard deviation (SD) if normally distributed, with comparisons between groups performed using independent samples *t*-tests. Non-normally distributed continuous variables are expressed as median and interquartile range (IQR), and compared using Mann-Whitney U tests. Categorical variables were compared using Chi-square tests. The discriminatory performance of individual variables for identifying metastatic LNs was assessed using ROC analysis. The area under the curve (AUC), sensitivity, specificity, and corresponding 95% confidence intervals (CI) were calculated. Comparisons of AUCs were performed using the DeLong test. Multivariable binary logistic regression analysis was conducted using the forward conditional method. Variables were entered into the model based on a significance level of *P* < 0.05 and removed if *P* > 0.10. Variables demonstrating a significant association (*P* < 0.05) in univariate analysis were considered for inclusion in the initial model pool. Results are reported as odds ratios (OR) with 95% CI. A multivariable diagnostic model was constructed based on the identified independent predictors. The overall discriminatory performance of this model was evaluated using ROC.

curve analysis, reporting the AUC and 95% CI. In all analyses, a two-tailed P value < 0.05 was considered statistically significant.

## Results

### Study population

The final cohort comprised 166 histopathologically confirmed cervical LNs. Figure 1 outlines the enrollment flowchart, illustrating the screening and exclusion process. Key demographic and clinicopathological characteristics of the patients and LNs are summarized in Table 1. Overall, the cohort displayed a balanced sex distribution and covered a representative range of nodal levels and sizes. Among metastatic LNs, lung cancer was the predominant primary origin.

**Table 1:** Patient and LNs baseline Characteristics.

Characteristic	
Participants (*n* = 166)	
Sex n (%)	
M	78 (47.0)
F	88 (53.0)
Age (years), median (IQR)	38 (32–48)
Level n (%)	
II	25 (15.1)
III	40 (24.1)
IV	70 (42.2)
V	31 (18.7)
Size (mm) median (IQR)	
Long axis	24 (13–28)
Short axis	13 (8–18)
surgical history n (%)	
No	90 (54.2)
Yes	76 (45.8)
Metastatic n (%)	77 (46.4)
Lung cancer	28 (36.4)
Thyroid cancer	15 (19.5)
Gastric cancer	14 (18.2)
Breast cancer	12 (15.6)
Other cancer	8 (10.4)
Reactive n (%)	89 (53.6)

### Quantitative SRUS parameter comparison

SRUS quantitatively revealed distinct microvascular signatures in metastatic LNs compared to their reactive counterparts. Metastatic LNs were characterized by significantly elevated vascular density, architectural complexity, and hemodynamic disorder, as detailed in Table 2. Specifically, parameters reflecting microvascular morphology, such as VD and FD, were substantially higher in the metastatic group. Hemodynamic parameters, including Vel Entropy and PI, were also markedly increased, whereas the Vmin was significantly reduced.

**Table 2:** Comparison of SRUS parameters between metastatic and reactive cervical LNs.

Parameter	Metastatic (*n* = 77)	Reactive (*n* = 89)	*p*-value
VD	0.482 ± 0.073	0.405 ± 0.168	< 0.001
Dmax	6.272 ± 1.626	6.054 ± 1.480	0.613
Dmean	2.229 ± 0.589	2.109 ± 0.614	0.565
Dstd	2.063 ± 0.537	2.013 ± 0.478	0.400
FWVD	1.784 ± 0.592	1.495 ± 0.813	0.013
FD	1.678 ± 0.070	1.626 ± 0.098	< 0.001
Vmean	25.758 ± 3.397	25.486 ± 4.393	0.013
Vmax	48.949 ± 7.694	49.472 ± 5.778	0.002
Vmin	2.572 ± 2.200	2.645 ± 2.800	0.017
Vel Var	149.761 ± 33.210	147.115 ± 53.164	< 0.001
Vel Entropy	0.922 ± 0.092	0.796 ± 0.199	< 0.001
Dir Var	4391.301 ± 1094.492	4383.695 ± 1355.021	0.048
Dir Entropy	0.730 ± 0.077	0.639 ± 0.122	< 0.001
PI	12.617 ± 2.563	10.369 ± 5.006	< 0.001

Data are presented as mean ± standard deviation. Abbreviations: VD, vascular density; Dmax, maximum vessel diameter; Dmean, mean vessel diameter; Dstd, vessel diameter standard deviation; FWVD, flow-weighted vascular density; FD, fractal dimension; Vmean, mean flow velocity; Vmax, maximum flow velocity; Vmin, minimum flow velocity; Vel Var, velocity variance; Vel Entropy, velocity entropy; Dir Var, direction variance; Dir Entropy, direction entropy; PI, perfusion index. P-values were derived from independent samples t-tests or Mann-Whitney U tests as appropriate.

**Fig. 3:**
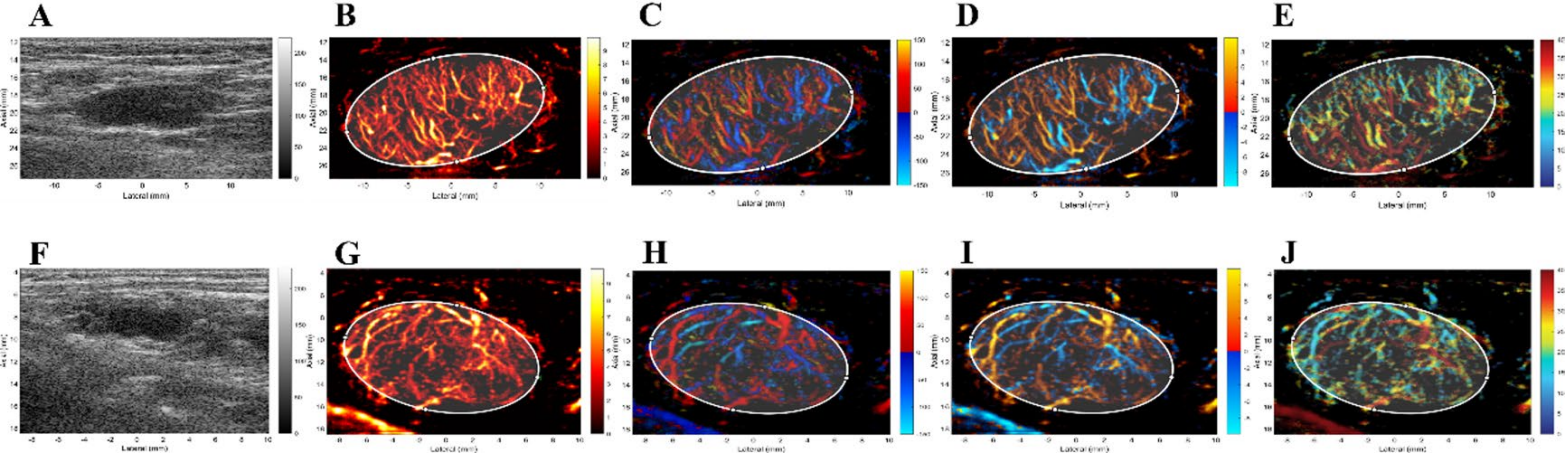
Representative SRUS images of reactive and metastatic LNs. Reactive LNs (**A**–**E**): (**A**) 2D-mode image showing an oval-shaped node with preserved fatty hilum; (**B**) Density map illustrating homogeneous microvascular distribution; (**C**) Direction map demonstrating organized directional flow; (**D**) Bi-direction map with symmetric bidirectional flow; (**E**) Velocity map revealing relatively uniform and elevated flow velocities. Example: A 35-year-old female with reactive hyperplasia; VD: 0.46; Vel Entropy: 0.72). Metastatic LNs (**F**–**J**): (**F**) 2D-mode image showing an oval-shaped node with preserved fatty hilum; (**G**) Density map displaying heterogeneous microvascularity; (**H**) Direction map showing disorganized and chaotic flow directions; (**I**) Bi-direction map with asymmetric and irregular bidirectional flow; (**J**) Velocity map revealing heterogeneous and low-velocity flow. Example: A 52-year-old male with lung cancer metastasis; VD: 0.49; Vel Entropy: 0.95).

These quantitative differences are visually corroborated by the parameter maps and representative SRUS images presented in Figs. 2 and 3. Figure 2 demonstrates the visibly elevated and more complex microvasculature in a metastatic LN across multiple parameters (PI, FWVD, FD, VD) compared to a reactive LN. Similarly, Fig. 3 illustrates the transition from a homogeneous, organized vascular network with uniform flow in reactive LNs (Figs. 3A-E) to a disorganized, dense network with chaotic flow in metastatic LNs (Figs. 3F-J).

### Diagnostic performance

In univariate analysis, several SRUS parameters demonstrated significant discriminatory power for identifying metastatic LNs (Table 3). VD and Dir Entropy were among the top individual performers, with AUCs of 0.689 and 0.723, respectively.

**Table 3:** Univariate diagnostic performance of SRUS Parameters.

Models	AUC	*p*	95%CI
VD	0.689	< 0.001	0.606–0.773
FWVD	0.656	0.001	0.572–0.741
FD	0.671	< 0.001	0.587–0.754
Vmean	0.524	0.595	0.435–0.612
Vmax	0.519	0.670	0.427–0.611
Vmin	0.468	0.476	0.379–0.557
Vel Var	0.561	0.176	0.470–0.651
Vel Entropy	0.675	< 0.001	0.590–0.760
Dir Var	0.497	0.955	0.409–0.586
Dir Entropy	0.723	< 0.001	0.646–0.799
PI	0.672	< 0.001	0.588–0.755

VD = vascular density; FWVD = Flow Weighted Density of the vessel; FD = Fractal dimension; Vmean = mean flow velocity; Vmax = maximum flow velocity; Vmin = minimum flow velocity; Vel Var = Velocity Variance; Vel Entropy = Velocity Entropy; Dir Var = direction variance; Dir Entropy = direction entropy; PI = Perfusion Index.

Multivariable logistic regression analysis refined these findings by identifying a combination of five independent predictors of metastasis (Table 4). The model highlighted the dominant role of morphological parameters like VD and PI, alongside key hemodynamic markers such as a decrease in Vmin and an increase in Vel Var and Vel Entropy.

**Table 4:** Multivariate logistic regression analysis to predict metastatic LNs.

	β	*p*	Exp(B)	95% CI
VD^1^	0.045	0.001	1.046	1.019–1.073
†FWVD	0.019	0.957	0.981	0.490–1.966
†FD	0.275	0.263	1.317	0.813–2.131
Vmean	-0.030	0.551	0.970	0.879–1.071
Vmax	0.008	0.776	1.008	0.954–1.066
Vmin	-0.644	0.003	0.525	0.342–0.806
Vel Var	0.679	0.016	1.973	1.211–3.485
†Vel Entropy	1.542	0.042	4.674	1.060-20.599
†Dir Var^1^	0.408	0.183	1.504	0.825–2.742
†Dir Entropy	0.483	0.281	1.621	0.674–3.903
PI	0.909	0.018	2.481	1.168–5.271

* VD^1^: Analyzed as percentage (×100); † FD, Vel Entropy, Dir Entropy, Dir Var, FWVD: Values were Z-standardized prior to analysis. β: regression coefficient; Exp(B): odds ratio; 95% CI: confidence interval.

**Fig. 4:**
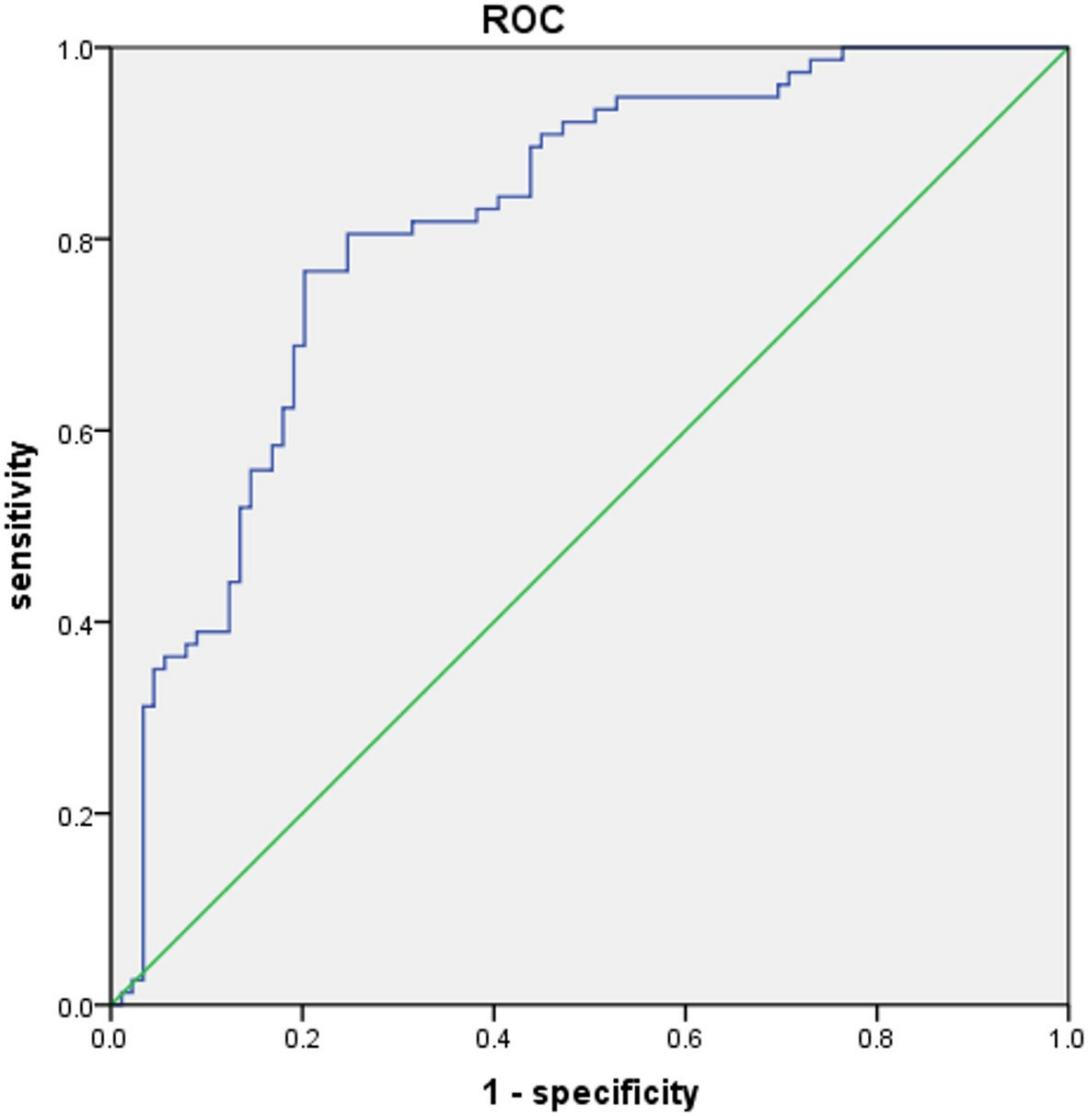
Receiver Operating Characteristic (ROC) Curve for the Multiparametric Diagnostic Model. The curve evaluates the model’s performance, which combines five independent SRUS parameters (VD, Vmin, Vel Var, Vel Entropy, and PI), in differentiating metastatic from reactive cervical lymph nodes. The model achieved an Area Under the Curve (AUC) of 0.813 (95% CI: 0.748–0.879).

The integrative power of this multiparametric approach is showcased in Fig. 4. The final model, which combined VD, Vmin, Vel Var, Vel Entropy, and PI, achieved superior diagnostic performance, with an AUC of 0.813 (95% CI: 0.748–0.879), a sensitivity of 76.6%, and a specificity of 79.8%.

## Discussion

This study demonstrated the significant potential of SRUS for the non-invasive characterization of microvascular morphology and hemodynamics within cervical LNs, enabling accurate detection of metastatic involvement, including micrometastases. Our key findings reveal distinctive SRUS signatures in metastatic nodes, including elevated VD, FD, PI, Vel Entropy, and Vel Var, alongside reduced Vmin. Crucially, a multivariable model combining VD, Vmin, Vel Var, Vel Entropy, and PI achieved excellent diagnostic performance (AUC = 0.813).

The observed parametric alterations align well with established pathological features of metastatic LNs. Elevated VD and FD reflect tumor-induced aberrant angiogenesis, histologically manifested as higher vessel density and disorganized branching patterns^14–17^. Increased Vel Entropy corresponds to turbulent flow within immature tumor vasculature, consistent with chaotic blood flow observed immunohistochemically in regions of high VEGF expression^18,19^. Reduced Vmin may relate to abnormalities within the tumor microvascular environment, such as leakiness due to deficient vessel maturation, microcirculatory disturbances, or heterogeneous flow distribution, phenomena consistent with pathological alterations commonly found in metastatic LNs^20,21^.

SRUS uniquely overcomes the inherent resolution limitations (~ 200 μm) of Doppler ultrasound, CT, and MRI^22,23^, enabling in vivo visualization of capillary-level pathological changes previously observable only via histopathology. Compared to the current diagnostic gold standard (i.e., surgical pathology with serial sectioning and IHC/RT-PCR), SRUS offers the advantages of being non-invasive and providing real-time functional information. SRUS also confers significant advantages over other key imaging modalities. It provides superior resolution (~ 10 μm vs. millimeter-scale for MRI) for quantifying microvascular hemodynamic details. Compared to PET/CT, SRUS avoids radiation exposure and demonstrates sensitivity in PET-negative metastatic foci (which constituted 30% of lesions included in this study). Relative to DCE-MRI, SRUS directly quantifies true microvascular hemodynamics (e.g., Vel Entropy), whereas DCE-MRI parameters primarily reflect large vessel characteristics and contrast leakage^24–26^. SRUS also offers advantages in portability, lower cost, and rapid bedside imaging.

Our results represent a significant advancement in the field. While SRUS microvascular quantification has been validated in animal models and hepatic imaging^27,28^, this is the first study to demonstrate its robust diagnostic utility for detecting metastatic involvement in human LNs. The identified elevation in Vel Entropy corroborates earlier reports of increased local flow direction irregularity^13^, solidifying abnormal hemodynamics as a key biomarker. Although CEUS improves the assessment of larger vessels, its inherent resolution limit (~ 100 μm) fundamentally hinders the reliable detection of early microvascular changes associated with micrometastasis^8^ – a limitation overcome by SRUS.

Based on our findings, we propose potential pathways for the clinical integration of SRUS: Risk Stratification: Apply SRUS to sonographically suspicious LNs; nodes with high model scores can be prioritized for biopsy. Biopsy Guidance: Target areas of hemodynamic abnormality identified by SRUS (e.g., regions of high Vel Entropy) to improve sampling yield for micrometastases. Treatment Monitoring: Utilize dynamic changes in parameters such as PI and Vel Entropy to assess response to anti-angiogenic therapies (e.g., bevacizumab).

This study has limitations. First, the single-center design carries a potential for selection bias; a prospective multicenter validation trial is planned for a later phase. Second, SRUS remains sensitive to motion artifacts; however, emerging deep learning-based motion correction algorithms show promise in preliminary testing, increasing usable image rates to 95%. Third, LNs with significant deformations such as calcification or cystic degeneration were excluded; future work should focus on optimizing SRUS sequences for such challenging scenarios and on the differential diagnosis of metastatic LNs versus other pathologies like lymphoma and granulomatous lymphadenitis, which represent common clinical dilemmas. The model requires validation in larger, more diverse populations. Technical refinements should include deep learning-based motion correction^29^ and 3D volumetric SRUS imaging. Future research should also explore correlations between SRUS parameters and molecular angiogenic markers (e.g., VEGF), evaluate the clinical utility of SRUS-guided targeted biopsy, and assess its impact on patient management decisions (e.g., treatment selection, follow-up strategies).

## Conclusion

SRUS imaging, leveraging its unique capability to quantify microvascular hemodynamics at micron-level resolution, represents a promising non-invasive tool for characterizing the underlying pathophysiology of cervical LNs. Key quantitative SRUS parameters effectively differentiate metastatic from reactive lymphadenopathy. The high diagnostic performance of our multiparametric model, derived from unique microvascular and hemodynamic insights beyond the resolution limits of conventional ultrasound, supports its potential role as an adjunctive tool for accurate early staging in head and neck cancer. This approach holds promise for optimizing biopsy decisions and refining patient management strategies by non-invasively delivering crucial functional insights.

## Data Availability

The datasets used and/or analyzed during the current study are available from the corresponding author on reasonable request.
